# Influence of Formulations on Characteristics of Ruthenium-Based Temperature-Sensitive Paints

**DOI:** 10.3390/s22030901

**Published:** 2022-01-25

**Authors:** Tsubasa Ikami, Koji Fujita, Hiroki Nagai

**Affiliations:** 1 Institute of Fluid Science, Tohoku University, Sendai 980-8577, Japan; fujita.koji@tohoku.ac.jp (K.F.); nagai.hiroki@tohoku.ac.jp (H.N.); 2 Department of Aerospace Engineering, Tohoku University, Sendai 980-8579, Japan

**Keywords:** temperature-sensitive paint (TSP), temperature sensitivity, pressure dependency, luminescent intensity, photodegradation, surface roughness

## Abstract

Temperature-sensitive paint (TSP) can optically measure a global temperature distribution using a thermal quenching of dye molecules. The TSP measurement is often used in wind tunnel tests to measure the temperature and flow fields in the aerodynamic field. The measurement accuracy is affected by the characteristics of TSP, such as temperature sensitivity, pressure dependency, luminescent intensity, photostability, and surface condition. The characteristics depend on the formulation of TSP. This study investigates the characteristics of the TSP using dichlorotris (1,10-phenanthroline) ruthenium(II) hydrate (Ru-phen). We compare the characteristics of TSPs using different polymers, solvents, and dye concentrations. The TSPs using polyacrylic acid as a polymer shows linear calibration curves, high luminescent intensity, high photostability, and smooth surface. On the other hand, the TSPs using polymethyl methacrylate have nonlinear calibration curves, low luminescent intensity, strong photodegradation, and a rough surface.

## 1. Introduction

Temperature is one of the most important physical quantities in fluid dynamics. Temperature-sensitive paint (TSP) is an optical measurement technology using thermal quenching of dye molecules. It is often used in wind tunnel tests [1]. Non-contact measurement is one of the advantages of TSP. Some probes such as a thermocouple or resistance thermometer are attached directly to test models. However, these attached probes can interfere with the flow field, and the presence of the probes would influence the measurement results in wind tunnel tests. On the other hand, TSP is applied to the surface of the test models with a thickness of several tens of µm. Moreover, the luminescent intensity is measured in a non-contact method since TSP is an optical method that uses fluorescence and phosphorescence of dye molecules. Therefore, TSP has less interference with the flow field than the attached probes. Furthermore, it is also possible to measure two-dimensional and high spatial resolution temperature distribution using CMOS or CCD cameras.

IR thermometry is also an optical measurement method of the temperature field, similar to TSP [2,3,4,5,6,7,8]. However, the measurement accuracy deteriorates in a cryogenic condition because the IR thermometry uses thermal radiation to measure the temperature. The cryogenic wind tunnels are used to reproduce high Reynolds number environments. Therefore, the temperature measurement in the cryogenic condition is important for evaluating the boundary layer transition in the research and development of aircraft. In addition, accurate measurement is also difficult in the water because part of the infrared radiation is absorbed by water. On the other hand, TSP can be applied even in cryogenic conditions or water since it uses thermal quenching, and the luminescence is the visible radiation. Therefore, TSP is also used for flow field measurement in cryogenic wind tunnels and water tunnels [9,10].

One use of TSP in experimental fluid dynamics is temperature correction for pressure-sensitive paint (PSP) [11,12]. PSP is also an optical measurement method of pressure field using oxygen quenching of dye molecules. However, the PSP has not only pressure sensitivity but also temperature sensitivity at the same time. The temperature sensitivity of the PSP is one of the error factors of pressure measurement [1]. Therefore, it is necessary to apply temperature correction to achieve a high accuracy measurement. TSP is often used for the correction because it is also an optical measurement method.

It is also possible to estimate the surface flow field on a test model from the temperature distribution measured by TSP. In the measurement of boundary layer transition using TSP, the laminar-to-turbulent boundary layer transition is visualized as a temperature difference using the difference in heat transfer coefficient between laminar and turbulent flows [9,13,14]. Boundary layer transition is directly related to the drag of aircraft and high-speed railways. Recently, TSP with carbon nano-tubes (CNT) thin heating layer, called cntTSP, has been developed [15,16]. The cntTSP has been used for the boundary layer transition measurement under various conditions, from cryogenic wind tunnels to low-speed wind tunnels [17,18,19,20].

Furthermore, a technique to measure skin friction using optical flow and TSP has been proposed in recent years [21,22,23]. Skin friction is one of the physical quantities that is difficult to measure in wind tunnel tests. Therefore, this measurement technique is valuable because it can evaluate skin friction quantitatively and two-dimensionally. However, it should be noted that the boundary layer transition and the skin friction are sensitive to the surface roughness of the test model. In TSP measurement, it is necessary to apply the paint to the surface of the test model. Thus, the surface roughness after the painting is required to be sufficiently smooth so that the TSP layer itself does not influence the measurement.

Moreover, it is also possible to calculate the heat flux and heat transfer coefficient distributions using the time-series TSP data [24,25,26,27,28]. Quantitative heat flux measurement is an important technique for developing hypersonic vehicles, which will experience significant aerodynamic heating [29].

TSP consists of a dye that shows temperature sensitivity, a polymer that adsorbs the dye on the surface of the test model, and a solvent that dissolves them. The desirable characteristics of TSP are high temperature sensitivity, high luminescent intensity, high photostability, and zero pressure dependency. Pressure sensitivity is caused by oxygen quenching of dye molecules. Therefore, pressure sensitivity can be suppressed by using a polymer with low oxygen permeability. In addition, photostability can be improved by using a polymer with low oxygen permeability since photodegradation proceeds in the presence of oxygen [30].

The temperature sensitivity and luminescent intensity are closely related to dye molecules. Ondrus et al. developed a europium complex (Europium 1,3-di (thienyl) propane-1,3-diones) with high temperature sensitivity and high luminescent intensity [31]. Furthermore, Egami et al. investigated the characteristics of TSP with various acrylic polymers and europium (III) thenoyltrifluoroacetonatetrihydrate (EuTTA), which is one of the typical TSP dye molecules [32]. They reported that the temperature sensitivity and luminescent intensity changed depending on the dye concentration and acrylic polymer. In other words, not only the dye molecule but also the polymer and dye concentration influence the characteristics of TSP.

Dichlorotris (1,10-phenanthroline) ruthenium(II) hydrate (Ru-phen) is also one of the typical TSP dye molecules. The lifetime of Eu(TTA) is about 500 µs, while that of Ru-phen is about 1 µs [1]. Therefore, Ru-phen is suitable for measurements that require high time resolution, such as shock wind tunnels with a millisecond order measurement duration [33,34,35,36]. Ru-phen also has the advantage of being excited by the visible blue light. However, the relationship between the characteristics of TSP and the formulation has not been sufficiently investigated in the Ru-phen based TSP. Therefore, in this study, Ru-phen was used as a dye, and the effects of polymer, dye concentration, and solvent on the characteristics of TSP were investigated. In addition, the surface roughness of TSP was also evaluated in this study, considering the applications to the boundary layer transition and skin friction measurements.

## 2. Principle of TSP

**Figure 1:**
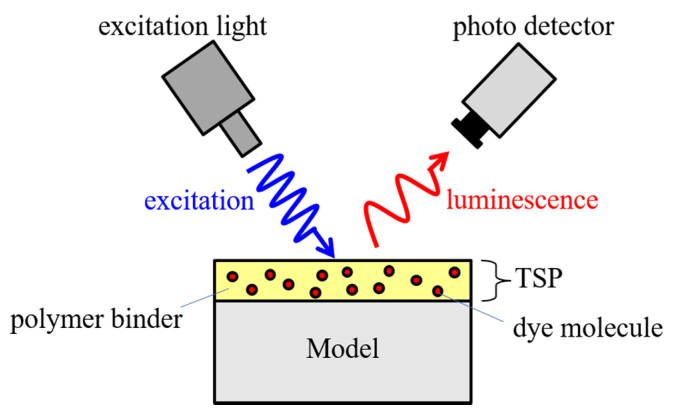
Principle and constitution of TSP measurement.

Thermal quenching is the fundamental principle in the TSP measurement [1]. Figure 1 shows the configuration of the TSP measurement. The TSP consists of a dye molecule that acts as a temperature sensor and a polymer binder that adsorbs the dye molecule. As a result, TSP forms a thin layer by painting on the model surface. The TSP layer is illuminated with excitation light of a specific wavelength. The dye molecule in the stable ground state transitions to the excited state by obtaining the energy of the excitation light. The excited dye molecules release energy and return to a stable ground state. This process is called deactivation. The deactivation process includes radiative deactivation with luminescence and non-radiative deactivation without luminescence. In radiation deactivation, the luminescent wavelength is longer than the excitation wavelength, called the Stokes shift. At high temperatures, collisions between molecules are more likely to occur. As a result, non-radiative deactivation increases, and radiation deactivation decreases. In other words, the luminescent intensity decreases with the increase of temperature. This relation is called thermal quenching. Therefore, the temperature can be calculated by measuring the change in luminescent intensity due to the thermal quenching with a photodetector such as a camera.

The relationship between the luminescent intensity and temperature can be written as the Arrhenius equation in Equation (1) [1].

(1) $$\ln\frac{I}{I_{ref}}=\frac{E}{R}\left(\frac{1}{T}-\frac{1}{T_{ref}}\right)$$

Here, *I* is the luminescent intensity, *T* is the temperature, *E* is the activation energy for the non-radiative process, *R* is the universal gas constant, and the subscript *ref* represents the reference state. In practice, a polynomial form in Equation (2) is often used instead of Equation (1).

(2) $$\frac{I}{I_{ref}}=\overset{N}{\underset{i=0}{\sum }}A_{i}{\left(\frac{T}{T_{ref}}\right)}^{i}$$

Here, *N* is the degree of the polynomial, and *A_i_* (*i* = 0, 1, …, *N*) are the polynomial coefficients. *A_i_* can be obtained in the calibration test.

## 3. Preparation of Test Piece

### 3.1. Formulations of TSP

This study investigated the characteristics of Ru-phen based TSP, and the dye concentration, polymer, and solvent were changed. The dye concentration *C* was defined by Equation (3).

(3) $$C\left(\mathrm{mg}/g\right)=\frac{\mathrm{mass}\;\mathrm{of}\;\mathrm{dye}\;\left(\mathrm{mg}\right)}{\mathrm{mass}\;\mathrm{of}\;\mathrm{polymer}\;\left(g\right)}$$

Table 1 summarizes the formulations of TSP investigated in this study. The dye concentration was changed considering the previous study that used Ru-phen [26]. Two types of polymer binders, polyacrylic acid (PAA) and polymethyl methacrylate (PMMA), were compared. They are typical polymers of TSP. Ethanol (EtOH) was used as the solvent, and Ru-phen was soluble. It should be noted that PAA is soluble in EtOH, but PMMA is insoluble. Therefore, in the formulations using PMMA, toluene or dichloromethane (DCM) was added to EtOH. Here, Ru-phen is insoluble in toluene and DCM. The mass of the polymer was constant at about 80 mg, and the dye concentration was controlled by changing the mass of the dye. For the paint preparation, the solvent, polymer, and dye were placed in a bottle with a cap. The bottle was stored in a dark room for a day to wait for dissolving the polymer and dye in the solvent.

### 3.2. Coating

**Figure 2:**
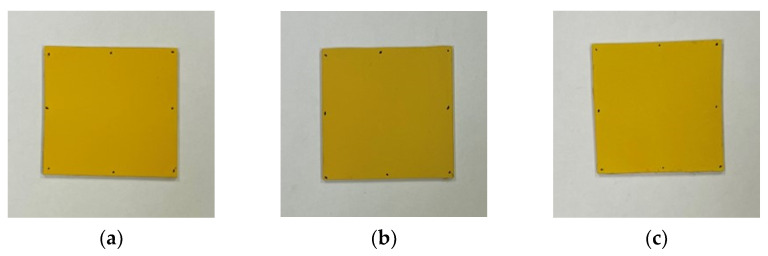
Pictures of TSP test pieces. (**a**) PAA + EtOH (*C* = 100.0 mg/g); (**b**) PMMA + EtOH + Toluene (*C* = 102.6 mg/g); (**c**) PMMA + EtOH + DCM (*C* = 85.4 mg/g).

The test piece was prepared by painting on an aluminum plate with a 40 mm × 40 mm and a thickness of 1 mm. First, white paint (MH12006, Musashi Holts, Tokyo, Japan) was applied as a white screen layer on an aluminum plate. The screen layer has the effect of reflecting the luminescent emission of TSP and increasing the intensity. Next, the TSP solutions were painted using an airbrush. After painting, the test piece was dried in a desiccator in a dark room for about 12 h. Figure 2 shows examples of the painted test piece. The black dots in Figure 2 are control points for position correction. The process from the paint application to the calibration test was completed within 24 h.

## 4. Experimental and Analytical Methods

### 4.1. Calibration System

**Figure 3:**
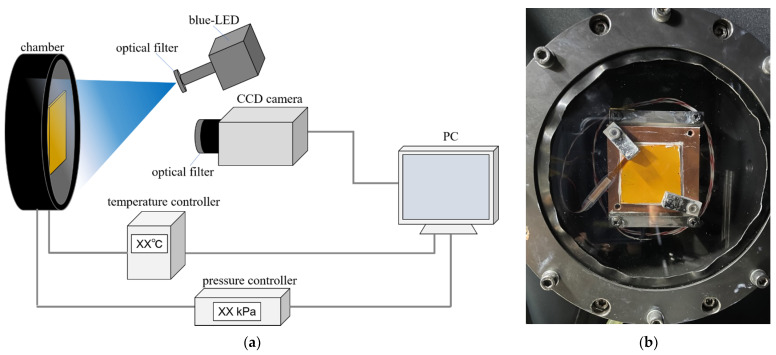
Pictures of a calibration system for TSP. (**a**) Schematic of components and their connections; (**b**) Picture of the chamber. Test piece is mounted in the chamber.

Figure 3 shows a schematic and photograph of the TSP calibration system used in this experiment. This calibration system can control the temperature in the range of 5–60 °C and the absolute pressure in 0–200 kPa. The pressure in the chamber was controlled by a pressure controller (PACE6000, Baker Hughes, Houston, TX, USA). The TSP test piece was placed on a Peltier unit (PU-50WS, Takagi MFG., Hitachinaka, Japan) in the chamber. The surface temperature of the TSP was measured by a resistance temperature detector (NFR-CF2-0305-20-100S-1-1000TF(PTFE13)-A-3-M4Y, Netsushin, Miyoshi, Japan). The TSP surface temperature was changed by feeding back to a temperature controller (TDU-5000A RG, Cell System Co., Ltd., Yokohama, Japan) and controlling the Peltier unit. The optical window of the chamber is made of quartz glass.

A blue LED (IL-106B LED, HARDsoft Microprocessor Systems, Kraków, Poland) was used as the excitation light source for the TSP. The central wavelength of this excitation light was 462 nm. A condensing lens (φ180 mm at 1 m) was attached to this light source, and a cold filter (SC0451, Asahi Spectra, Tokyo, Japan) and a heat ray absorption filter (HAF-50 S-30 H, SIGMA KOKI, Hidaka, Japan) were installed in front of the condensing lens. The distance between the excitation light and the test piece was 420 mm. The current of the excitation light source was set to 18 A, and the image acquisition was started after 10 min of warm-up operation. The test piece was covered to prevent exposure to the excitation light during the warm-up operation. A 16-bit CCD camera (C4742-98 KAG2, Hamamatsu Photonics, Hamamatsu, Japan) was used to detect the luminescent emission of TSP. A lens (Nikkor 50 mm f/1.4, Nikon, Tokyo, Japan) was attached to this camera, and an optical filter (575 nm CWL, 50 mm Dia. Hard Coated OD 4.0 50 nm Bandpass Filter, Edmund optics, Barrington, NJ, USA) was installed in front of the lens.

### 4.2. Calibration Test

The calibration test was performed using the calibration system described in Section 4.1. The CCD camera acquired images with changing the TSP’s surface temperature in the range of 5–50·°C. The pressure was applied under two conditions of 80 kPa and 100 kPa to check the pressure dependency.

In the analysis, the luminescent intensity was averaged in the same 128 × 128 pixels range for each test piece. The averaged values were used as the luminescent intensity under the temperature and pressure conditions. In addition, the standard deviation of the luminescent intensity in the averaged range was shown in the graph as an error bar. In addition, images were obtained at the same temperature and pressure at the beginning and end of the calibration test. The photodegradation rate was estimated by comparing these two images, and the luminescent intensity was corrected using the photodegradation rate.

The local temperature sensitivity *S_T_* was calculated by Equation (4) [32,37].

(4) $$S_{T}\;\left(\%/℃\right)=-\left(\frac{dI\left(T\right)}{dT}\right)\frac{1}{I\left(T\right)}\times 100$$

The luminescent intensity data were interpolated by spline. The temperature sensitivity was calculated by differentiating the interpolated data as shown in Equation (4).

### 4.3. Measurement of Luminescent Intensity

The luminescent intensities of each test piece were compared with different formulations. The temperature and pressure were set to 20 °C and 100 kPa, respectively. The image acquisition conditions of the camera and the output of the excitation light source were the same for all the test pieces. The luminescent intensities were averaged in the same 128 × 128 pixels area for all the test pieces. The averaged values were defined as the luminescent intensities. The standard deviations in the averaged area were defined as the error bars.

### 4.4. Measurement of Photodegradation

The test piece was continuously illuminated with the excitation light source. The decrease in luminescent intensity due to photodegradation was evaluated. The temperature and pressure were constant at 20 °C and 100 kPa, respectively. Images were acquired every minute for 1 h after the start of illumination. The luminescent intensities in 128 × 128 pixels were averaged for each test piece for the acquired images. The standard deviation of the luminescent intensity in the averaged area was shown in the graph as an error bar.

### 4.5. Measurement of Surface Condition

The surface roughness was measured using a surface roughness measuring device (HANDYSURF + 35 E-MD-S217A, TOKYO SEIMITSU, Hachioji, Japan). The surface roughness was measured at any 5 points on each test piece with a length of 4 mm each, and the arithmetic average roughness *Ra* was measured. The bar graph represents the median, and the maximum and minimum values are represented by error bars.

## 5. Results and Discussion

### 5.1. Temperature Sensitivity

This section discusses the results obtained in the calibration test. The horizontal axis shows the temperature, and the vertical axis shows the luminescent intensity in the calibration curve. The luminescent intensity was standardized by the intensity at a temperature of 20 °C and a pressure of 100 kPa. The pressure was kept constant at 100 kPa. The temperature sensitivity was calculated from the calibration curve using Equation (4).

#### 5.1.1. PAA + EtOH

**Figure 4:**
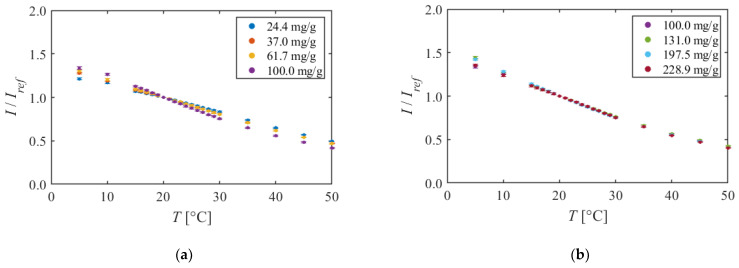
Calibration curves of PAA + EtOH in different dye concentrations. The reference condition is *T* = 20 °C and *P* = 100 kPa. (**a**)The dye concentration is 100 mg/g or less. (**b**)The dye concentration is 100 mg/g or more.

Figure 4 shows the calibration curves of PAA + EtOH. Figure 4a is the calibration curve with 100.0 mg/g or less dye concentration. The luminescent intensity decreased linearly for temperature for all dye concentrations. Moreover, the decrease slope was steeper as the dye concentration increased from 24.4 mg/g to 100.0 mg/g. Figure 4b is calibration curves with 100.0 mg/g or more dye concentration. The qualitative change was not observed in the calibration curves; even the dye concentration changed from 100.0 mg/g to 228.9 mg/g.

**Figure 5:**
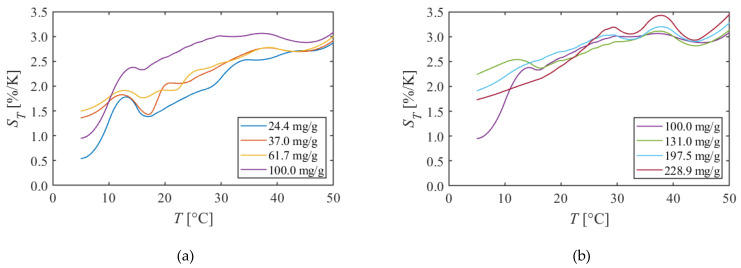
Temperature sensitivities of PAA + EtOH in different dye concentrations. (**a**) The dye concentration is 100 mg/g or less. (**b**) The dye concentration is 100 mg/g or more.

Figure 5 shows the temperature sensitivity of PAA + EtOH calculated from Figure 4. Figure 5a is the temperature sensitivity of the dye concentration of 100.0 mg/g or less. It was observed that the temperature sensitivity was higher as the dye concentration increased in the range of 100.0 mg/g or less. Specifically, the temperature sensitivity was 1.6%/K when the dye concentration was 24.4 mg/g; however, it increased to 2.6%/K when the dye concentration was 100.0 mg/g at 20 °C. On the other hand, there was no qualitative change in temperature sensitivity due to changes in dye concentration of 100.0 mg/g to 228.9 mg/g, and it was about 2.5%/K at 20 °C, as shown in Figure 5b. Previous studies have reported a temperature sensitivity of the Ru-phen based TSP, which uses PAA as a binder, was about 1.7–2.2%/K around room temperature, and that consists with the results of this study [26].

PAA + EtOH has a temperature sensitivity of about 2.5%/K around 20 °C. It is a sufficient temperature sensitivity in wind tunnel tests that requires detecting temperature changes of 2–3 °C. Furthermore, a linear calibration curve is observed in the range of 5–50 °C. Therefore, it can be concluded that this TSP is applicable in a wide temperature range.

#### 5.1.2. PMMA + EtOH + Toluene

**Figure 6:**
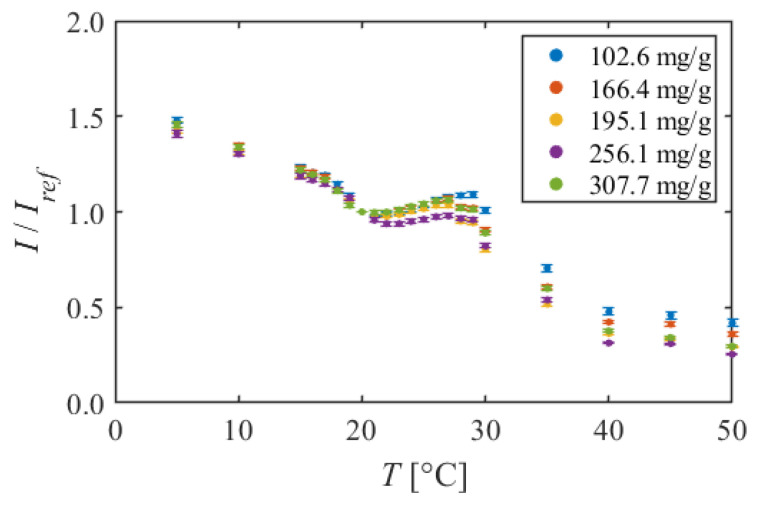
Calibration curves of PMMA + EtOH + Toluene in different dye concentrations. The reference condition is *T* = 20 °C and *P* = 100 kPa.

**Figure 7:**
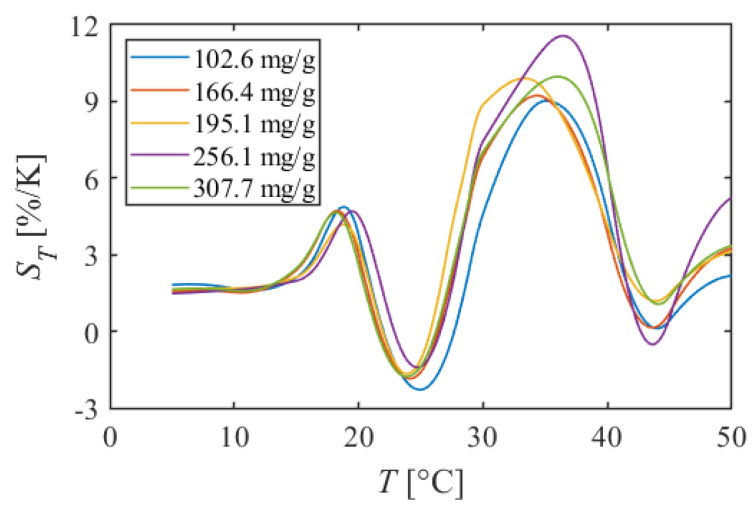
Temperature sensitivities of PMMA + EtOH + Toluene in different dye concentrations.

Figure 6 shows the calibration curve of PMMA + EtOH + Toluene, and Figure 7 shows the temperature sensitivity. The calibration curves had nonlinear characteristics compared to PAA + EtOH for all dye concentration conditions. At 5–18 °C, the luminescent intensity decreased linearly with temperature. In this temperature range, temperature sensitivities were about 1.6%/K. However, at 18–21 °C, the luminescent intensity decreased sharply, and the temperature sensitivity increased to 4.5%/K. At 21–29 °C, the luminescent intensity increased slightly despite the increasing temperature. Therefore, the temperature sensitivities took negative values and decreased to about −2.3%/K at 25 °C at 102.6 mg/g. In the higher temperature range of 29–40 °C, the luminescent intensity dropped sharply again. In this temperature range, the temperature sensitivity reached 11.5%/K at 36 °C at 256.1 mg/g. Finally, at 40–50 °C, the decrease in luminescent intensity became gentle, and the temperature sensitivity was about 3%/K.

PMMA + EtOH + Toluene has a nonlinear calibration curve compared to PAA + EtOH. Especially around room temperature of 20–30 °C, there are some points where the calibration curve does not decrease monotonically with temperature. It can be concluded that this TSP is unsuitable for use as a temperature sensor around these points since the temperature cannot be determined uniquely for the luminescent intensity. On the other hand, at 30–40 °C, the TSP shows a very high temperature sensitivity of more than 10%/K. It can be said that this TSP is a high sensitivity temperature sensor only in this temperature range.

#### 5.1.3. PMMA + EtOH + DCM

**Figure 8:**
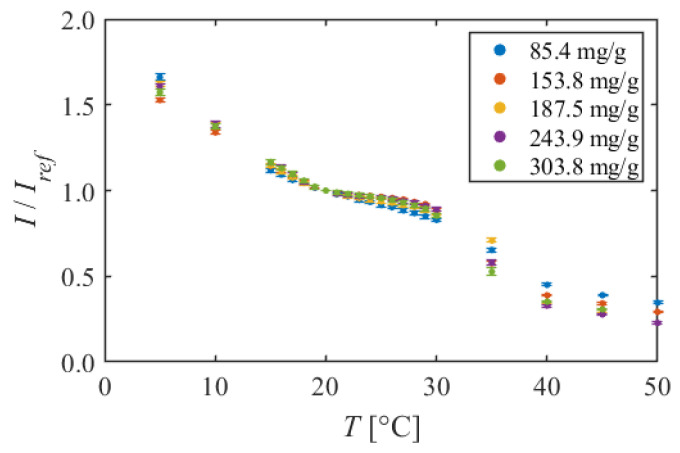
Calibration curves of PMMA + EtOH + DCM in different dye concentrations. The reference condition is *T* = 20 °C and *P* = 100 kPa.

**Figure 9:**
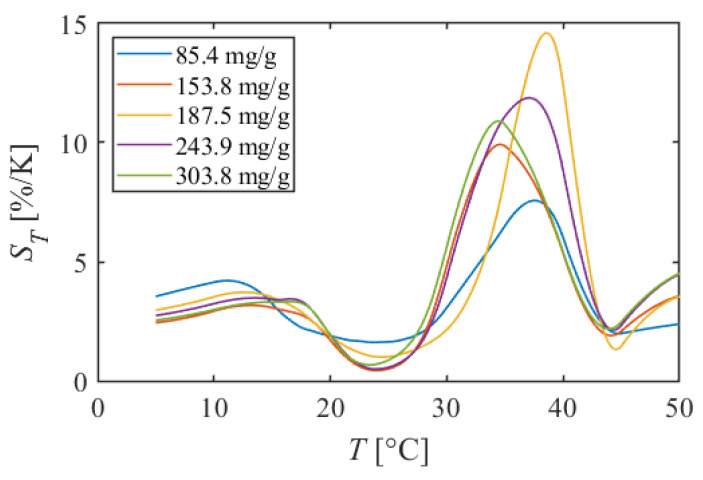
Temperature sensitivities of PMMA + EtOH + DCM in different dye concentrations.

Figure 8 shows the calibration curves of PMMA + EtOH + DCM, and Figure 9 shows the temperature sensitivity. At 5–20 °C, the luminescent intensity decreased linearly with increasing temperature. The temperature sensitivities were about 3%/K in this temperature range. Next, nonlinear curves were observed around 20 °C. The slope of the calibration curve was gentler at 85.4 mg/g, and the temperature sensitivity decreased at 20–30 °C. However, the temperature sensitivity was 1.6%/K in the dye concentration of 85.4 mg/g, even at the lowest point. While, at the dye concentration of 153.8–303.8 mg/g, the calibration curves were almost flat, and the temperature sensitivities were low, for example, 0.5%/K at 24 °C in the dye concentration 243.9 mg/g. In PMMA + EtOH + Toluene, the temperature sensitivity took a negative value, but in PMMA + EtOH + DCM, the temperature sensitivities kept the positive values. At 30–40 °C, the luminescent intensity decreased sharply, and the high temperature sensitivities were observed for all dye concentrations, such as 14.4%/K at 39 °C in the dye concentration of 187.5 mg/g. In the higher temperature range of 40–50 °C, the decrease in luminescent intensity became gentle again, and the temperature sensitivity became about 3%/K.

This TSP also has nonlinear calibration curves compared to PAA + EtOH. However, the temperature sensitivity keeps positive values. In addition, at around room temperature, the temperature sensitivity is 1.6%/K at the low dye concentration of 85.4 mg/g. Moreover, very high temperature sensitivity is observed at 30–40 °C. It can be said that this TSP is a highly sensitive temperature sensor only in this temperature range.

### 5.2. Pressure Dependency

**Figure 10:**
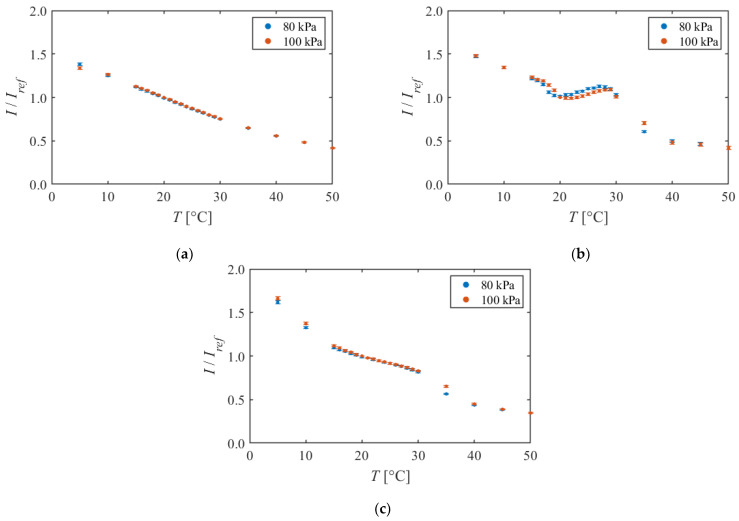
Calibration curves in different pressure conditions. The reference condition is *T* = 20 °C and *P* = 100 kPa. (**a**) PAA + EtOH (*C* = 100.0 mg/g); (**b**) PMMA + EtOH + Toluene (*C* = 102.6 mg/g); (**c**) PMMA + EtOH + DCM (*C* = 85.4 mg/g).

Figure 10 shows the pressure dependence of each formulation. The temperature was changed in the range of 5–50 °C, and the luminescent intensity was compared between two pressure conditions, 80 kPa and 100 kPa. In Figure 10, the dye concentration is about 100 mg/g for all formulations. The luminescent intensities are standardized with *I_ref_*, which is the luminescent intensity at a temperature of 20 °C and a pressure of 100 kPa.

In Figure 10a PAA + EtOH, the difference was very small, and the luminescent intensities showed good agreement between 80 kPa and 100 kPa. For example, at 20 °C, the intensity at 100 kPa was 0.84% higher than the intensity at 80 kPa. This difference was smaller than the error bar and negligible. In Figure 10b PMMA + EtOH + Toluene, there was no pressure dependency in the low temperature region of 5–15 °C and the high temperature region of 40–50 °C. However, the luminescent intensities were different between two pressure conditions in the temperature region of 15–40 °C, where a nonlinear tendency was observed in the calibration curves. In Figure 10c PMMA + EtOH + DCM, no change in the luminescent intensity was observed due to changes in pressure around room temperature of 20–30 °C. On the other hand, pressure dependence was observed in the range of 5–15 °C and 30–40 °C, where the slope of the calibration curve was steep.

The pressure sensitivity of TSP is one of the error factors of temperature measurement. Therefore, the pressure sensitivity of TSP should be zero. Therefore, it can be concluded that PAA + EtOH is superior to the other two formulations from pressure dependence.

### 5.3. Luminescent Intensity

**Figure 11:**
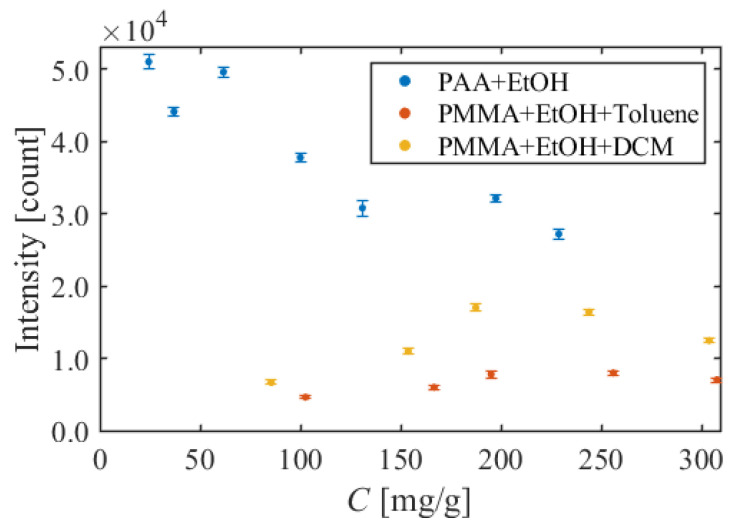
Comparison of luminescent intensity in different dye concentrations and formulations.

Figure 11 shows the luminescent intensity with the different dye concentrations for each formulation. In Figure 11, the horizontal axis is the dye concentration, and the vertical axis is the count of a 16-bit CCD camera. The luminescent intensity increases with the dye concentration in the range of the dye concentration under the specific value. However, when the dye concentration exceeds a specific value, the luminescent intensity decreases due to concentration quenching [38]. For PAA + EtOH, the luminescent intensity decreased as the dye concentration increased in the investigated dye concentration range. Therefore, it can be concluded that the concentration quenching occurred in this range. Next, for PMMA + EtOH + Toluene and PMMA + EtOH + DCM, the luminescent intensity increased with the dye concentration in the range of less than 200 mg/g. The luminescent intensity decreased slightly at more than 200 mg/g, but no clear peak was confirmed in this experiment.

The luminescent intensities were also different among these three formulations. Comparing the luminescent intensity at the dye concentration of about 100 mg/g (PAA + EtOH: 100.0 mg/g, PMMA + EtOH + Toluene: 102.6 mg/g, PMMA + EtOH + DCM: 85.4 mg/g), PAA + EtOH was 3.8 × 10^4^ count, PMMA + EtOH + Toluene was 0.5 × 10^4^ count, and PMMA + EtOH + DCM was 0.7 × 10^4^ count. In the same way, the luminescent intensity was higher in the order of PAA + EtOH > PMMA + EtOH + DCM > PMMA + EtOH + Toluene for other dye concentrations. It was found that PAA + EtOH has the highest luminescent intensity of the three formulations even considering the concentration quenching.

### 5.4. Photodegradation

**Figure 12:**
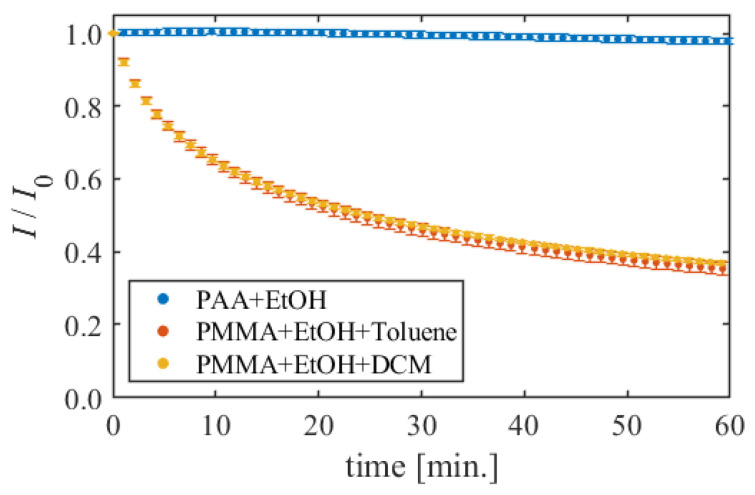
Comparison of photodegradation in different dye concentrations.

Figure 12 shows the time-series luminescent intensity in the photodeterioration test. The dye concentration for each formulations is about 100 mg/g (PAA + EtOH: 100.0 mg/g, PMMA + EtOH + Toluene: 102.6 mg/g, PMMA + EtOH + DCM: 85.4 mg/g). The luminescent intensity was standardized by the intensity at the measurement start time in each formulation.

First, for PAA + EtOH, the photodegradation hardly progressed, and the luminescent intensity decreased by 2.1% in 1 h. On the other hand, the photodegradation progresses in formulations using PMMA, and the intensity decreased 65.0% for PMMA + EtOH + Toluene, and 63.4% for PMMA + EtOH + DCM.

Photodegradation is also one of the error factors in TSP measurement [1]. PAA + EtOH has good photostability, and the influence of photodegradation on measurement accuracy can be small even in long-term measurement. However, PMMA + EtOH + Toluene and PMMA + EtOH + DCM are greatly influenced by photodegradation. Therefore, the measurement time should be shortened, or correction should be conducted for photodegradation to assure measurement accuracy.

### 5.5. Surface Roughness

**Figure 13:**
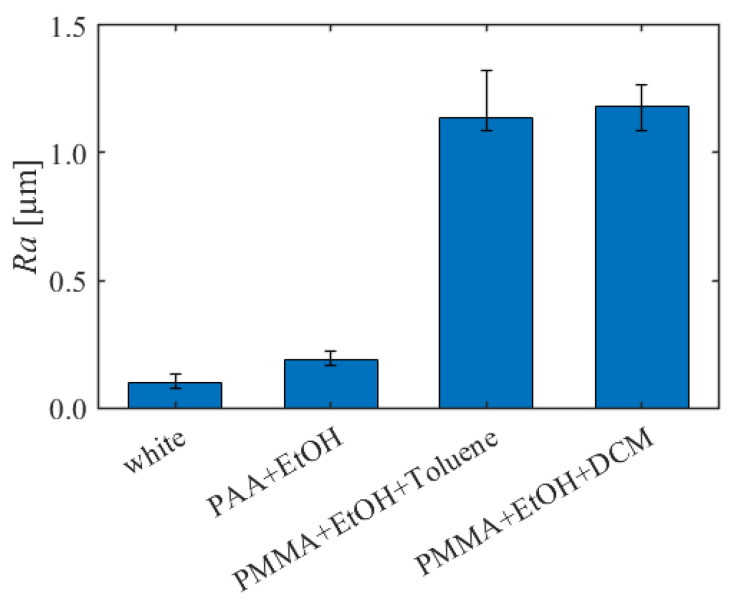
Comparison of surface roughness in different dye concentrations.

Figure 13 shows the surface roughness of the test pieces. The dye concentrations were about 100 mg/g for all formulations (PAA + EtOH: 100.0 mg/g, PMMA + EtOH + Toluene: 102.6 mg/g, PMMA + EtOH + DCM: 85.4 mg/g). “White” represents the surface roughness of the white screen layer. The surface roughness increased by coating TSP for all formulations. The surface roughness was 0.189 µm for PAA + EtOH, while it increased to 1.134 µm for PMMA + EtOH + Toluene and 1.180 µm for PMMA + EtOH + DCM, respectively. Therefore, it was found that a smoother surface can be provided by using PAA as the binder compared to PMMA.

## 6. Conclusions

We investigated various characteristics of Ru-phen based TSP, such as temperature sensitivity, pressure dependence, luminescent intensity, photodegradation, and surface roughness. The influence of the polymer, dye concentration, and solvent on these characteristics was also investigated. The findings obtained for the three formulations are summarized as follows:

- PAA + EtOH

This formulation had a linear calibration curve over a wide temperature range of 5–50 °C. It showed a temperature sensitivity of 2.5%/K at 20 °C. In addition, there was no pressure dependence. Moreover, it showed better photostability, higher luminescent intensity, and smoother surface roughness than the other two formulations using PMMA.

- PMMA + EtOH + Toluene

This formulation showed a nonlinear calibration curve. In particular, the luminescent intensity increased even though the temperature increased around room temperature of 20–30 °C. In addition, there was a dependency of luminescent intensity on the pressure around 18–40 °C, and the photodegradation progressed significantly compared to PAA + EtOH. Moreover, this formulation had a rougher surface than PAA + EtOH. The luminescent intensity was the lowest among these three formulations.

- PMMA + EtOH + DCM

The nonlinear calibration curves were also observed in this formulation, but it showed monotonic decreases with temperature. There was a slight change in luminescent intensity due to pressure in the low and high temperature ranges. Furthermore, this formulation also showed significant photodegradation compared to the formulation using PAA. In addition, the surface was rougher than PAA + EtOH. The luminescent intensity was higher than that of PMMA + EtOH + Toluene. However, it was about 1/4 that of PAA + EtOH at the same dye concentration.

## Figures and Tables

**Table 1:** Summary of formulations investigated in this study. Three kinds of formulations were investigated.

Formulation	Polymer (Mass)	Solvent (Volume)	Dye Concentration
PAA ^1^ + EtOH ^2^	PAA ^1^ (80 mg)	EtOH ^2^ (6 mL)	24.4–228.9 mg/g
PMMA ^3^ + EtOH ^2^ + Toluene	PMMA ^3^ (80 mg)	EtOH ^2^ (2 mL) + Toluene (4 mL)	102.6–307.7 mg/g
PMMA ^3^ + EtOH ^2^ + DCM ^4^	PMMA ^3^ (80 mg)	EtOH ^2^ (2 mL) + DCM ^4^ (4 mL)	85.4–303.8 mg/g

^1^ PAA: polyacrylic acid; ^2^ EtOH: ethanol; ^3^ PMMA: polymethyl methacrylate; ^4^ DCM: dichloromethane.

## Data Availability

Data sharing not applicable.
